# Exploring the perceptions of faculty members about research courses in undergraduate pharmacy curriculum: A qualitative study

**DOI:** 10.1371/journal.pone.0305946

**Published:** 2024-06-25

**Authors:** Aaliah Aly, Ola Hishari, Derek Stewart, Ahmed Awaisu, Sara Elshami, Banan Mukhalalati

**Affiliations:** Department of Clinical Pharmacy and Practice, College of Pharmacy, QU-Health, Qatar University, Doha, Qatar; Endeavour College of Natural Health, AUSTRALIA

## Abstract

**Introduction and objective:**

The commitment of pharmacy graduates to patient care and research is crucial to advancing pharmaceutical science and practice. Consequently, the value of involving undergraduate pharmacy students in research has been increasingly recognized. Given that the College of Pharmacy at Qatar University offers two undergraduate pharmacy research courses, it is relevant to explore the perception of faculty members of the delivery, impact, facilitators, barriers, and suggested improvements in these courses. This exploration will help to improve the existing curriculum and to highlight the prospective impact of student involvement in undergraduate pharmacy research courses on the personal and professional growth of students, as well as on the progressive evolution of the pharmacy profession.

**Methods:**

A qualitative exploratory case study was performed in which five virtual focus groups were conducted. All eligible faculty members from the clinical pharmacy and pharmaceutical science departments with experience supervising students who had taken one or two undergraduate pharmacy research courses were invited to participate. The focus group questions were based on the Theoretical domain framework of behavioral determinants. Verbatim transcription was performed, and the collected data were thematically analyzed using the computer-assisted coding software NVivo®.

**Results:**

Of the 26 eligible faculty members, 21 participated in this study. Five deductive themes were identified: social professional role and influences, beliefs about capabilities, skills, beliefs about consequences and goals, and environmental resources and behavioral regulations. Overall, faculty members identified themselves as assessors and mentors. Participants expected students to possess independence, responsibility, and motivation. They believed that students generally required more practical research skills. Several benefits of incorporating students into undergraduate research have been highlighted, including increased publication productivity and quality. However, several hurdles to undergraduate research in pharmacy have been identified, including limited resources, limited timeframes, and sometimes delayed ethical approval.

**Conclusion:**

Faculty members expressed optimism regarding the undergraduate research courses. However, some logistical concerns, including the lengthy ethical approval process and resource availability, must be addressed to optimize the effectiveness of these courses.

## Introduction

In recent decades, the pharmacy profession and the scope of its professional practice have evolved [1]. This transformation in pharmacy practice requires competent and committed pharmacists who can conduct pharmacy practice research (PPR) to guarantee the implementation of evidence-based practice (EBP) [2]. However, despite the significant role of PPR in advancing practice, the literature suggests that the involvement of pharmacists in PPR falls short of the demands of professional practice, hindering the application of EBP [3]. Worldwide, numerous studies have investigated the existing barriers associated with the suboptimal involvement of pharmacists in research. A systematic review indicated that most pharmacists did not receive adequate education and training in PPR during their undergraduate degree programs, which negatively affected their competence in planning and conducting research [3]. Other key obstacles to providing the necessary research experience to pharmacy students were reported, such as inadequate implementation of research courses in pharmacy curricula [4], lack of time [5–8], and shortage of research mentors [4].

Many universities around the world are currently undergoing significant reforms in pharmacy education [9]. This educational reform aims to equip pharmacy students and graduates with the necessary competencies to conduct research and apply research findings to professional practice [10, 11]. Furthermore, it aligns well with the “nine-star pharmacist” concept, which was introduced by the World Health Organization and the International Pharmaceutical Federation, acknowledging that a well-rounded pharmacist should possess the following qualities: a care giver, life-long learner, good decision maker, communicator, and manager with leadership characteristics, entrepreneur, constant learner, and have teaching skills and research abilities [12]. This educational reform aligns with both the Accreditation Council for Pharmacy Education (ACPE) guidance and one of the core competencies outlined by the Institute of Medicine, which aims to equip students with robust research and clinical skills to enhance patient care and engage them in scholarly pursuits [13, 14]. Key points of this guidance include emphasizing the integration of research methods across the curriculum, encouraging active student involvement in research, and facilitating faculty participation in scholarly activities, all geared towards fostering a culture of inquiry and evidence-based practice within pharmacy education.

Several pharmacy colleges in the Eastern Mediterranean Region have pursued international pharmacy accreditation/certification [15] by one of the following agencies: the Australian Pharmacy Council (APC), German Accreditation Agency in Health and Social Sciences, Canadian Council for Accreditation of Pharmacy Programs (CCAPP), and Accreditation Council for Pharmacy Education (ACPE). Research and scholarship experience are integral components of the accreditation standards for BSc pharmacy programs set by the APC and CCAPP [16, 17]. The CCAPP elaborated on the skills that students should acquire to conduct PPR should include being able to identify relevant problems; generate a hypothesis; design a study; analyze data; interpret and apply findings; disseminate research findings; and apply regulatory and ethical principles [18].

Several pharmacy schools and colleges offer undergraduate research courses worldwide [19, 20]. Previous studies have indicated that these courses enhance students’ confidence in conducting research [21, 22]. Furthermore, early exposure to research training allows students to increase their pharmaceutical and clinical knowledge and expertise by enhancing their abilities to define a problem, systematically collect and analyze data, and make informed decisions [22].

The College of Pharmacy at Qatar University (CPH-QU) is the only pharmacy college accredited by the CCAPP in Qatar [23]. CPH-QU focuses on developing students’ research skills by offering six compulsory Pharmacy Research Evaluation and Presentation skills (PREP) courses and two undergraduate pharmacy research courses (UPRCs) throughout its five-year undergraduate program. The UPRCs are offered as an elective course during the third (P3) professional year and as a mandatory course during the fourth (P4) professional year of the BSc Pharmacy degree program. Therefore, all students are exposed to at least one practical research experience [23]. However, there is a scarcity of research exploring the faculty members’ perceptions of the facilitators and barriers of UPRCs. Therefore, using select domains of the Theoretical Domain Framework, version 2 (TDFv2) [24], this study aimed to 1) examine the perceptions of faculty members about the delivery and impact of undergraduate research courses in CPH, 2) examine the perceptions of faculty members regarding facilitators and barriers to the delivery and impact of undergraduate research courses, and 3) examine possible improvements to undergraduate research courses according to the perceptions of faculty members. These objectives were pursued with the goal of improving the existing curriculum through elucidating various facilitators and barriers associated with UPRCs and emphasizing the potential influence of student involvement in UPRCs on the personal, and professional development of students and on the pharmacy practice.

## Materials and methods

### Study design and setting

This study was conducted at CPH-QU from January to April 2022. A qualitative exploratory case-study approach was utilized [25]. Data collection, analysis, and interpretation were guided by the constructivist interpretative framework, which focuses on processes and contexts, considering the subjective and social negotiation of meanings while acknowledging the influence of researchers’ values [26]. This study was conducted in accordance with the Consolidated Criteria for Reporting Qualitative (COREQ) Studies guidelines [27].

### Study population and eligibility criteria

All faculty members from both the Clinical Pharmacy and Practice (CPP) and the Pharmaceutical Sciences (PSC) departments at CPH-QU, who have been involved in supervising students who had taken one or two URPCs since the inception of the college (n = 26), were eligible to participate in this study. Including faculty members from both departments was important to recognize potential differences in perspective and expertise across these distinct research areas.

An invitation email was sent to all eligible faculty members. The email included a consent form and a participant information leaflet with details of the study methods, confidentiality, and anonymity measures, as well as the anticipated benefits and risks.

### Data collection tool

A topic guide was developed by two researchers, AA, and OA, under the supervision of the rest of the research team, utilizing the TDFv 2. The complete topic guide is available in S1 File. The TDFv2 comprises a series of theoretical domains assist in developing and effectively assessing facilitators and barriers to implementing interventions [24]. In the context of the present study, employing the TDFv2 can systematically investigate behavioral determinants associated with the successful delivery and implementation of UPRCs from the perspective of faculty members, shedding light on areas of strength and areas needing improvement. Additionally, the framework enables the identification of potential strategies and interventions to address the identified barriers and enhance the delivery and impact of these courses.

The topic guide included nine of the fourteen TDF domains: skills, professional role and identity, social influences, emotion, beliefs about capabilities, beliefs about consequences, goals, environmental context and resources, and behavioral regulation. To identify the most relevant TDFv2 Domains, the topic guide questions were initially aligned with all available domains. Subsequently, through the process of refining the topic guide questions, certain domains emerged as capable probes for exploring other domains more relevant domains, while others appeared to be less pertinent. This recognition was achieved through consensus among members of the research team. Table 1 illustrates the rationale behind including and excluding the TDF domains in this study.

**Table 1:** Description and rationale for including and excluding specific TDFv2 domains.

Domain	Description	Rationale
**Included domains**		
Professional role and identity	A coherent set of behaviors and displayed personal qualities of an individual in a social or work setting	To investigate the current and anticipated roles and responsibilities necessary for both faculty members and students to attain the optimal benefit derived from UPRCs These three domains were merged into one theme. This integrated theme considered the professional role and identity or the internal self-concept of faculty members and the social influences involving the role of students
Social influences	Those interpersonal processes that can cause an individual to change their thoughts, feelings, or behaviors	
Beliefs about capabilities	Acceptance of the truth, reality, or validity about an ability, talent, or facility that a person can put to constructive use	To identify faculty members’ perceptions of their own and their students’ capabilities, which act as facilitators or barriers to conducting UPRCs
Skills	An ability or proficiency acquired through practice	To investigate the skills necessary for faculty members and students to possess to successfully conduct UPRCs is essential
Beliefs about consequences	Acceptance of the truth, reality, or validity about outcomes of a behavior in a given situation	To understand the faculty members’ intentions and beliefs about consequences and to highlight perceived benefits and drawbacks and emphasizing the importance and impact of UPRCs All domains were merged into one theme. Understanding the linkage between domains can help in identifying what drives the faculty members’ commitment to teaching research courses and how they measure success
Goals	Mental representation of outcomes or end states that an individual wants to achieve	
Optimism	The confidence that things will happen for the best or that desired goals will be attained	
Intentions	A conscious decision to perform a behavior or a resolve to act in a certain way	
Environmental context and resources	Any circumstance of a person’s situation or environment that discourages or encourages the development of skills and abilities, independence	To investigate environmental factors (like institutional support or constraints) and individual actions (like time management and prioritization) affect the successful conduction of UPRCs
Behavioral Regulation	Anything aimed at managing or changing objectively observed or measured actions	
**Excluded domains**		
Knowledge	An awareness of the existence of something	It would be inappropriate to inquire about the faculty’s knowledge of research courses. These courses have been consistently offered for many years, and the faculty members possess extensive experience in research
Memory, attention, and decision process	The ability to retain information, focus selectively on aspects of the environment and choose between two or more alternatives	The research process does not rely on memorizing information. Each type of research follows a specific protocol or guideline that is readily accessible.
Emotion	A complex reaction pattern, involving experiential, behavioral, and physiological elements, by which the individual attempts to deal with a personally significant matter or event.	Given that these courses are of a scientific nature, emotions and personal feelings are less likely to influence their conduction
Reinforcement	Increasing the probability of a response by arranging a dependent relationship, or contingency, between the response and a given stimulus	UPRCs are part of the pharmacy undergraduate curriculum, therefore, the research team found reinforcement irrelevant to inquire about, as it was expected that all faculty members would maintain professionalism and avoid letting their personal experiences influence their role as supervisors

The nine selected TDF domains were distributed among the three sections of the topic guide; the first section focused on the delivery and impact of UPRC, the second focused on the associated barriers that hinder the optimal implementation of UPRCs, and the third focused on the suggested improvements of UPRCs.

### Data collection and analysis

Five focus groups were conducted. All focus groups were conducted virtually in English via Webex video in the period between 15^th^ February to 15^th^ of April 2022. All participants sent their signed consent before the beginning of the focus group. Each session lasted 60–90 minutes, was recorded and transcribed verbatim by one researcher, and the transcription accuracy was verified by a second researcher.

Deductive thematic analysis was used for data analysis using computer-assisted coding software, NVivo®, version 12.0.0.71. In which, the collected data underwent mapping to the priorly selected TDFv2 domains to facilitate comprehensive interpretation and analysis. To ensure the rigor of the data analysis, two researchers independently conducted a thematic analysis, convened to discuss discrepancies, and agreed to the final coding. Afterward, the participants were asked via email to verify that the transcripts and emerging themes accurately reflected their perspectives.

### Ethical considerations

Ethical approval was obtained from the Institutional Review Board of Qatar University (approval number: QU-IRB 1562-EA/21). The study adhered to the trustworthiness criteria of Lincoln and Guba for the quality assurance of a qualitative study (Table 2) [28].

**Table 2:** Measures to attempt trustworthiness criteria of Lincoln and Guba.

Measure	Application
**Credibility**	
Peer review	All steps were supervised by the peer-researcher and the research supervisors.
Multiple sources of data	Data was collected from five different FGs including faculty members from both CPP and PSC departments.
Member-checking	Proper transcription and interpretation of data was ensured through cross-checking between OA and AA. Thereafter, transcripts were returned to participants to check the accuracy of transcription. All respondents agreed with data analysis and interpretation.
**Dependability**	
Full description	Structured, detailed, and full description of the methodology was written out.
Data triangulation	Data collected from five different FGs.
Peer review	A peer researcher and research supervisor conducted audits for all the research steps.
Inter-coding reliability testing	The coding and the data analysis were done by two independent researchers, OA & AA.
Intra-coder reliability testing	Re-coding for the same data by the same researcher on two different time periods. The results of coding and recoding matched.
**Conformability**	
Full description of methodology	Structured, detailed, and full description of the methodology was written out to be followed.
Research process oversight	All steps were supervised by the research supervisors
Multiple sets of evidence	Data were collected from five different FGs
**Transferability**	
Recording the research details	Structured and detailed description about the settings, contexts, and methods of the FG is provided.
Triangulation	Data Triangulation: Five FGs including faculty members from both CPP and PSC departments were conducted. Investigator Triangulation: Two independent researchers collected and analyzed the data.

## Results

Of the 26 eligible faculty members who supervised the students’ research projects, 21 participated in this research. Six faculty members were from the PSC department and 15 were from the CPP department. Five themes were generated deductively using the TDFv2.

### Theme 1. Social professional role and influences

This theme focused on the perspective of faculty members regarding the current and anticipated roles and responsibilities necessary for both faculty members and students to attain the optimal benefit derived from UPRCs.

#### Subtheme 1.1. The role of faculty members as assessors and mentors

Several faculty members described their roles as assessors and mentors, as they are responsible for performing frequent assessments and providing feedback to guide the students accordingly. For example, a participant stated:

“The supervisor needs to have a quick assessment of the students who is joining to identify the extent the student has grasp on the theoretical parts of the project that is being proposed.” [Participant 5, PSC]

Another participant reinforced:

“As a mentor, you guide their thought process moving from a junior to a senior at the level of an undergraduate.” [Participant 2, CPP]

Concurrently, several participants illustrated that successfully supervising students in UPRCs requires faculty members to provide emotional and psychological support along the scientific guidance. In that regard, a faculty member stated:

"We have to ensure that we are there for them, not just in terms of the theory and science aspects of what they are doing, but also to give them some emotional support." [Participant 9, PSC]

Furthermore, empowering students to build confidence in their ideas and perspectives was brought up when discussing the anticipated professional role of faculty members while supervising students. A faculty member stated:

“Supervisors have to empower the students to do their own thing. So, that they have the initiative and eventually build their confidence to voice their ideas.” [Participant 10, CPP]

#### Subtheme 1.2. The role of students as responsible, independent, and motivated learners

Almost all participants agreed that students should be committed and hold the responsibility of doing tasks assigned to them on time to achieve the UPRCs’ learning outcomes. For instance, a faculty member said:

“The most important thing I would expect is independence and commitment to the excellence in whatever they are going to do in their best ability and responsibility” [Participant 5, PSC]

Another CPP faculty member stated:

“We are their mentors, but they are also the ones responsible for getting to know how those procedures are being done, of course under our supervision.” [Participant 3, CPP]

Additionally, faculty members emphasized on the importance of students to be self-motivated to use their abilities and be independent. A faculty member stated:

“I do not want the students to just be a follower, just telling them what to do. Instead, they should be self-driven to use their abilities, critical thinking, and analytical processes, and to put all the efforts independently to come up with the essential tasks” [Participant 5, PSC]

### Theme 2. Beliefs about research capabilities

The second theme focused on the perceptions of faculty members of their own and of the students’ capabilities and competencies, which serve as facilitators and barriers to conducting UPRCs.

#### Subtheme 2.1. The expertise of faculty members in research

There was an agreement among faculty members that their expertise in conducting enabled the effective supervision of undergraduate research projects. In this context, an interviewee stated:

"I would say, we have been doing research for many years and we have built the required set of skills and knowledge to supervise students, even if we stumble upon obstacles, because you know research is dynamic, we always find a way out." [Participant 9, PSC]

Another faculty member mentioned:

“We have strong confidence in our research expertise. This strong confidence enhances students’ enthusiasm and belief on the importance of research conduction, which is our primary goal at the end.” [Participant 21, CPP]

Whereas few faculty members reported that the occasional involvement of another principal investigator or a research assistant to guide students in using software, as needed. For example, a faculty member said:

“If I were to choose something, that is a bit more complex as a software, then I might add another colleague as a PI to this project, so that they can also help and better explain and guide the student.” [Participant 1, CPP]

#### Subtheme 2.2. The knowledge, skills, and experience of students in research

The good theoretical foundation and knowledge of students was highlighted by many faculty members as one of the facilitators of the UPRCs. A participant mentioned:

"I would say that students have a really strong foundation in the different research designs and methods, because of the PREP courses they finished before taking the UPRCs." [Participant 13, CPP]

However, few faculty members believed that PREP courses were theoretical and that students needed more practical research experience before taking UPRCs. For example, a faculty member said:

"Although students were introduced to different research methodologies, but when it comes to application, it was sometimes difficult due to lack of hands-on experience." [Participant 21, CPP]

Another PSC faculty member added:

“Initially, students may not be well qualified to conduct the required experiments by themselves due to lack of experience, and they need to be taught.” [Participant 4, PSC]

Moreover, it was highlighted by several participants that students who were previously enrolled in an Undergraduate Research Experience Program (UREP) or in PHAR445 had a better knowledge and skills compared to other students. For instance, a faculty member stated:

“All students have the basic knowledge required. However, fortunate students are those who were involved in a UREP project or PHAR445, before they get into to 545. They have a better baseline and confidence.” [Participant 6, CPP]

### Theme 3. Skills

The third theme discussed the skills and attributes that faculty members believe they, as faculty members and students, should possess to supervise students in UPRCs successfully.

#### Subtheme 3.1. The management and organizational skills of faculty members and learners

Several participants indicated that both faculty members and students should have organizational and management skills to facilitate the progress of the research effectively and efficiently. A faculty member said:

"Students along with us should possess time management skills, because this will facilitate a good progression in the course." [Participant 13, CPP]

Another faculty member added:

“Time management and organizational skills are important to be able to carry out this project.” [Participant 10, CPP]

Moreover, faculty members indicated that URPCs are delivered over one semester, therefore, students should manage and allocate time to finish the required work. An interviewee said:

“Students have to manage their time and allocate certain hours throughout their schedule for that project, because it is a lot of work to be done in a short time frame.” [Participant 10, CPP]

#### Subtheme 3.2. The communication and adaptability skills of faculty members and students

Several faculty members emphasized the importance of good communication skills to facilitate a collaborative relationship among faculty, students, and other researchers. In this regard, a faculty member stated:

“Good communication skills help in facilitating easy communication between the supervisor and the students and plays an important role to set up a baseline for the project to move ahead.” [Participant 5, PSC]

A faculty member added:

“Having good communication skills will make you as a supervisor able to engage with your students, and with other researchers, which will facilitate the research process.” [Participant 10, CPP]

Moreover, most faculty members highlighted the importance of flexibility and conflict resolution skills to adapt to unplanned changes during the research process. One participant stated:

"A very important skill for the supervisor is the ability to modify the scope of the project and limit the scope of the work in the event that the student is struggling." [Participant 20, CPP]

Moreover, faculty members emphasized on the necessity of accepting their assigned students regardless of their level of knowledge and skills. A faculty member stated:

"Patience and acceptance are things that you would need to develop, because not all students are going to be at the same skill level as others in conducting different aspects of the project." [Participant 3, CPP]

Another faculty member reinforced:

"I would say that we have to be patient and keep the expectations and it and enjoy the development that they would see in these undergraduate students" [Participant 5, PSC]

Another faculty member added that students also need to possess flexibility:

"Students are also needed to be flexible, listen to time management advice, and have the level of the emotional intelligence to deal with the difficult path of the project" [Participant 6, CPP]

### Theme 4. Beliefs about consequences & goals

The fourth theme focused on the beliefs of faculty members of the consequences of implementing UPRCs and on the individual, institutional, and national impacts of UPRCs.

#### Subtheme 4.1. Exposing students to hands-on research experience

Faculty members intensively described how engaging students in URPCs provided students with practical research experience. For example, a faculty member said:

“These research courses have been designed in such a way that they are providing the practical experience of implementing the theoretical knowledge they gained in PREP courses.” [Participant 5, PSC]

Another faculty member added:

“Our goal is that we want them to understand and apply the stages of the research process” [Participant 6, CPP]

Participants clarified by saying that enrolling students in UPRCs help them in developing research competencies. A faculty member mentioned:

“In UPRCs, they learn how to conceptualize research into ideas and how to turn these ideas into research questions… … also, it teaches them about the appropriate methodologies to handle data and to avoid any bias.” [Participant 11, PSC]

#### Subtheme 4.2. Increasing the publication productivity and quality

Several participants emphasized that UPRCs help faculty members get more publications and give the students the opportunity to publish. For instance, a participant said:

“There are positive consequences for us as faculty members, and as you can see, the majority of our projects in UPRCs get published, and it is positive for us as faculty to have work published." [Participant 7, CPP]

Another faculty member added:

“Students while they are still in their undergraduate years can have sometimes a publication, which makes them proud since they are still young in their career life” [Participant 10, CPP]

Several faculty members indicated that involving students in UPRCs can result in higher quality research and publication. For instance, one participant mentioned:

"Sometimes they bring out questions that actually direct the project in a more impactful area, and I think that this is the beauty of doing research with vibrant, young, energetic people, which results in higher quality and more impactful publications." [Participant 2, PSC]

#### Subtheme 4.3. Enhancing the reputation of the college

Faculty members indicated that delivering UPRCs has demonstrated nationally, regionally, and internationally how QU-CPH students are involved in research, which is a competitive advantage for CPH-QU. A participant stated:

"This will further show nationally and regionally how students are involved in research at a very early stage of their career, during their undergraduate studies" [Participant 1, CPP]

Another faculty member added that QU graduates are well recognized for their critical thinking and management skills, which enabled them to succuss in this course, and which added to the college to be distinguished. A participant stated:

“QU graduates are well known regionally for being prepared throughout their study years to develop critical thinking skills and other attributes that enable them to excel in these research courses. For instance, leadership skills, management skills. Students are known to be competent, and entrepreneurs.” [Participant 10, CPP]

#### Subtheme 4.4. Improving the scope of pharmacy practice in the country

At the national level, almost all participants agreed that involving students during their undergraduate in research is crucial in preparing them to practice. A faculty member mentioned:

“We’re preparing them for practice; research is a crucial component of what they will do in practice.” [Participant 11, CPP]

Furthermore, several faculty members indicated that URPCs have eventually improved the scope of pharmacy practice in Qatar. A faculty member said:

"Research conducted in UPRCs have looked into ways to improve pharmacotherapy and local patient outcomes by improving processes and preventing errors or adverse reactions." [Participant 10, CPP]

Another faculty member added:

"In UPRCs students are accomplishing small parts of large projects. So, the combined effect of all those parts of the project, could definitely, contribute a lot to the science, pharmacy practice, and might improve some aspects of policies in the country." [Participant 9, PSC]

### Theme 5. Environmental resources and behavioral regulations

The last theme focused navigating the environmental resources and behavioral regulations which tend to influence the facilitation or hindrance of UPRC conduction.

#### Subtheme 5.1. The availability of resources

Several faculty members highlighted the lack of the required software for certain research methodologies, as one of the barriers to conducting UPRCs. For example, a participant said:

"We conduct a number of qualitative research, but the software required for qualitative research are not provided by the university." [Participant 10, CPP]

Also, faculty members from both departments indicated that the support provided by the university in terms of hiring research assistants or lab technicians is not adequate. They added that this area needs improvement. For instance, a participant stated:

"We do not really have research assistants or lab technicians who would help us increase the research landscape. This needs to be improved." [Participant 9, PSC]

Moreover, several participants mentioned the “time” as one of the restrictions to achieving the optimal outcomes of UPRCs. A participant mentioned:

"Unfortunately, the undergraduate courses run over a very short period; 3–4 months. So, it is difficult sometimes to involve students in every single process of research." [Participant 3, CPP]

Therefore, participants suggested dedicating more time to these courses:

"Perhaps we need to revisit the way we have designed our curriculum and give more dedicated time for research and at least having the first or last two months only dedicated to the research course with no disruptions from other courses." [Participant 20, CPP]

Finally, the funding of research courses was highlighted by almost all the participants as one of the limitations of URPCs. A faculty member stated:

"The budget is a huge barrier that needs improvement, because when you receive a grant, it is only available for 2–3 months, which is extremely short period" [Participant 3, CPP]

#### Subtheme 5.2. The logistical aspects of research conduction in UPRCs

Ethical approval was recognized by the participants as one of the logistical barriers that may negatively affect the progression of research projects. A participant said:

"I think the QU Institutional Review Board approval is time-consuming, which makes us sometimes conduct projects that do not require approval." [Participant 10, CPP]. Therefore, several faculty members suggested having an ethics committee for undergraduate students at the college level to facilitate and the ethical approval.

Furthermore, several faculty members highlighted the process of random allocation of students to UPRCs as one of the limitations. A faculty member said:

"Random allocation of students for projects is not related to the interest of those students in the project, which might negatively impact their involvement." [Participant 1, CPP]

Other participants suggested that students get a choice in selecting their research topic:

"I think that students should get a choice in their projects. I think that this the main way to improve the UPRCs, because it helps to maintain students’ interests." [Participant 19, PSC]

Finally, faculty members stated that the extensive workload on them and on students due to their packed schedules is another barrier to conducting UPRCs. A faculty member said:

“URPCs are courses amongst a very busy schedule, so sometimes the student is wanting to commit but is bombarded with so many assessments, the midterms and so on. And same goes for us as faculty members, we need to teach, make exams and grade” [Participant 10, CPP]

## Discussion

This study aimed to align with the significance of developing and implementing academic programs that promote student engagement, foster critical thinking, ensure academic success, and ultimately prepare students for EBP, which depends on their ability to research and use the best available data to guide their decisions. Given a batch size of approximately 30 students, the URPCs are generally offered as an elective during the third professional year (P3). At this stage, half of the student batch can opt to enroll in the URPC or choose another elective. However, in the fourth professional year (P4), completion of the URPC becomes mandatory for all students. A recent study conducted within the same educational institute, CPH-QU, highlighted the substantial number of students enrolled in the UPRCs from the college’s inception until 2021 (n = 287) [29]. This highlights the necessity of adjusting the research curriculum to ensure that students receive comprehensive exposure to research methodologies and practices. Maximizing the educational experiences of these students is essential, as inadequate research training would represent a significant lost opportunity in their professional development and their ability to contribute effectively to the field of pharmacy.

The findings indicate that most participants from both departments elucidated the importance of their roles as assessors and mentors. This finding is congruent with a study conducted in 2015, which indicated that students predominantly expect their supervisors to mentor and guide them in acquiring the necessary research information and skills and then evaluate their work and provide constructive feedback [30]. Moreover, qualitative research published in 2019, with the aim of examining the role of supervisors’ responsibilities in research projects, revealed that research supervisors are expected to provide emotional and academic support and emphasized the importance of empowering students throughout their research [31]. Therefore, these findings highlight the importance of conducting an initial supervisor-student meeting to clarify the roles and responsibilities, thereby facilitating the successful completion of the UPRCs.

Regarding interpersonal skills, all participants identified organizational and management skills as essential when supervising research students in UPRCs. The participants elaborated that faculty members should formulate the research aim and methodology, develop reasonable expectations and timeframes, and follow up plans. They also indicated that supervisors should possess conflict-resolution skills to overcome potential obstacles. These findings align with Siddiqui and Jonas-Dwyer’s study, which highlighted the significance of the managerial and organizational skills of faculty members, along with conflict resolution, before they embark on supervising research students [32]. Several participants agreed that both supervisors and supervisees should have good communication and flexibility skills; this result is supported by the conclusion of a mixed-methods study by Ivankova and Stick that highlighted the importance of these skills in dealing with any unforeseen changes, particularly within the limited timeframe of research courses [33]. Hence, a broad range of competences are needed for the facilitation of effective research conduction, including interpersonal skills like communication, flexibility, and conflict resolution in addition to technical knowledge.

In this study, all participants expressed confidence in supervising students in UPRCs, which can be explained by their extensive research and supervision experiences. This finding is consistent with the that of qualitative research that suggested that experienced supervisors felt more comfortable and able to gauge the abilities of students than less experienced supervisors [34]. On the contrary, few faculty members who recently joined CPH-QU indicated their need for support from other faculty members, teaching assistants, or research assistants, which is consistent with a recent study from Yanbu University that highlighted the significance of mentoring novice supervisors to ensure high-quality undergraduate research experiences [35]. So, addressing the technical needs of faculty members could significantly enhance the quality of undergraduate research experiences. This, in turn, could lead to improved mentorship and eventual success of UPRCs.

Regarding the roles of students, most participants agreed that students are expected to be committed, responsible, passionate, and self-motivated. They further highlighted the significance of these characteristics. In this regard, it is worth noting that the results reported by the earlier study conducted by Mukhalalati et al., revealed significant self-motivation, interest and enthusiasm among students for research [29]. This is consistent with Lee’s finding that the responsibility, independence, and commitment attributes of students are crucial for a successful research process, as perceived by supervisors [36]. Moreover, a study that aimed to assess the opinions of Hungarian faculty members and the attributes of students required to optimally conduct research indicated that students are favored in showing commitment, motivation, and self-dependency [37]. Hence, acknowledging and highlighting the expectations placed on students can contribute to the optimization of the students’ research experience and outcomes.

Another key finding of this study is the strong theoretical foundation of students’ research before enrolling in UPRCs because of their prerequisite enrollment in PREP courses. Participants indicated that the enrollment of students in the two UPRCs exposes them to practical research experience, preparing them for future careers. This is reinforced by a 2016 study that investigated the factors influencing student gains from undergraduate research experiences. This study concluded that skill-building opportunities, such as workshops, seminars, and preparatory courses, are highly beneficial and increase students’ chances of gaining skills [38]. Therefore, students’ enrollment in preparatory courses contributes to the establishment of a solid knowledge foundation, which facilitates a better experience in UPRCs.

Nearly all participants believed that sustaining and advancing undergraduate research initiatives had a positive impact on students, faculty members, academic institutions, and the country. Participants believed that UPRCs provided students with an opportunity to gain full practical research experience, which is essential for their future careers and EBP. This finding is in line with those of a Malaysian study that concluded that undergraduate medical students perceive their research courses as opportunities to conduct practical research [39]. Similar results were demonstrated in a study on undergraduate research in medicine that concluded that undergraduate research is essential for the development of essential skills for students to enhance their clinical practice [40]. Furthermore, the findings of this research suggest that enrollment in UPRCs enhances student confidence in conducting research, which positively impacts their future careers as researchers. This aligns with the findings of a recent publication by Mukhalalati et al. in 2022, which emerged from the same institution, CPH-QU. It presents the perspective of students, illustrating that they believe UPRCs have enhanced their interest and confidence in conducting research [29]. This finding is also supported by cross-sectional research at King Saud University, which illustrated that more than 70% of pharmacy students who participated in undergraduate research courses were interested in pursuing a career in research [41]. Therefore, research skills, confidence, and career aspirations are shaped through practical research experiences from UPRCs.

For faculty members, participants explained that their involvement in UPRCs improved their publication productivity because students often participated in writing the research as a journal manuscript or conference paper. This finding aligns with the conclusion of two studies published in 2008 and 2022, which indicated that, as a consequence of supervising students, faculty members could either initiate or continue a productive research agenda [40, 42]. This finding also aligns with the results of Mukhalalati et al. (2022) study which found that over 50% of students enrolled in UPRCs published at least one peer-reviewed article, and nearly 70% of students submitted at least one abstract for a local or international conference, either as a poster or oral presentation [29]. Hence, the collaboration between faculty members and students in UPRCs is encouraged among faculty members as it is anticipated to increase scholarly output for both faculty members and students.

Regarding the impact of UPRCs on colleges, most faculty members indicated that incorporating UPRCs into the curriculum has positively impacted the reputation of the college and has become one of its competitive advantages. It is worth noting that providing students with research experience is one of the CCAPP requirements, as indicated in the following statement, "The pharmacy program must include a mission that encompasses professional education, scholarship, research, clinical practice, and professional service” [17]. Furthermore, research application is one of the educational outcomes of the Association of Faculties of Pharmacy of Canada (AFPC) and one of the Canadian National Association of Pharmacy Regulatory Authorities (NAPRA) competencies that students are expected to acquire prior to graduation [43–45]. Abiding by the CCAPP standards, AFPC and NAPRA competencies have helped the CPH-QU receive its Canadian accreditation by CCAPP and demonstrate its academic distinction in the region. Therefore, the integration of UPRCs not only enhances the educational experience for students but also contributes to the overall academic distinction and accreditation status of the college. It highlights the commitment of the educational institution to providing comprehensive and high-quality education that prepares students for successful careers in pharmacy practice and research.

At the national level of pharmacy practice, participants stressed that equipping undergraduate students with research experience aids in developing their decision-making, critical thinking, time management, and communication skills, which will ultimately enhance the scope of practice in Qatar. This finding is supported by an article published in 2022 by Mass-Hernández et al. that indicated that enrolling students in undergraduate research equips them with important skills that will be reflected during their careers; this, in turn, facilitates the development of the medical industry and practice nationally and globally [40]. Hence, integrating research experiences into undergraduate education not only benefits students and faculty members at their professional level but also has broader positive implications for the healthcare system.

The results of this study highlighted several barriers that need to be addressed, such as the availability of resources, money, and time. Additionally, despite a general student-faculty ratio of approximately 2:1 for research supervision (which varies depending on the number of enrolled students and the complexity of the proposed research projects) a significant shortage of research personnel to supervise the increasing number of students was noted. This finding is supported by a study conducted by Kiyimba et al. in 2022 that concluded that insufficient resources, especially funds, are a significant limitation to optimal research [46]. Furthermore, according to Assar et al. and Ferdoush et al., a lack of time, money, and personnel are the most prevalent barriers [47, 48]. Lastly, this study showed that logistics in terms of ethical approval is sometimes an obstacle to the effective delivery of UPRCs. This finding was also highlighted by Hart et al. who showed that lengthy ethical approval processes hinder undergraduate research [49]. This insight is crucial for researchers and institutions involved in planning and implementing UPRCs, as addressing these barriers is essential for ensuring the success and effectiveness of UPRCs.

As with any other research, this study has some limitations. This study utilized focus groups for data collection, which may have hindered participants from fully expressing their opinions. Therefore, incorporating semi-structured interviews with faculty members in future research will help address this limitation. Finally, while the exclusion of some TDF domains could have potentially led to missing important aspects of faculty members’ experiences in supervising UPRCs students, this decision was made to maintain focus and coherence within the analysis.

## Conclusions

This qualitative study was the first to evaluate the perceptions of CPH-QU faculty members regarding the delivery, impact, facilitators, barriers, and suggested improvements in UPRCs. This study demonstrated the effectiveness of understanding the behavioral determinants associated with the success of UPRCs from the perspective of faculty members. The results revealed that the faculty members interviewed in this study were broadly optimistic about the delivery and impact of UPRCs. However, there are concerns regarding the implementation of UPRCs in terms of ethical approval and the availability of resources requiring prompt intervention to ultimately enhance the overall effectiveness of UPRCs.

Future longitudinal studies to track changes in the perspectives of faculty members and identify the long-term impact of initiatives proposed by faculty members in this research may be warranted for continuous quality assurance and improvement of the UPRCs and, ultimately, of the pharmacy curriculum, so that graduating students can act as competent researchers. Moreover, despite quantitative research being conducted two years ago to understand the perceptions of pharmacy students and alumni toward research after completing URPCs at CPH–QU, complementing the findings with qualitative research with current student researchers is warranted. This deeper exploration of students’ perceptions will facilitate a thorough examination of areas where the current research program aligns or misaligns with student expectations, hence informing curriculum revisions and improvements. Finally, future research could explore the commonalities or differences across different Pharmacy colleges in different countries in the region. This can identify regional and context-specific best practices, enrich theoretical frameworks, and inform more effective and credible educational policies and practices, ultimately enhancing the quality of Pharmacy education in the country.

## Supporting information

S1 File Topic guide for faculty members’ focus group. (PDF)
