# Supplementary material for: Exploring the perceptions of faculty members about research courses in undergraduate pharmacy curriculum: A qualitative study
Source: PLoS One. 2024 Jun 25;19(6):e0305946. doi: 10.1371/journal.pone.0305946 (PMC11198811; doi:10.1371/journal.pone.0305946)
Supplement: S1 File — (PDF) [file pone.0305946.s001.pdf]

S1 File. Topic Guide for Faculty Members' Focus Group.

Research Questions:

- What are the perceptions of faculty regarding the delivery and impact of undergraduate research courses in CPH on students and faculty members?
- What are the perceptions of faculty regarding the facilitators and barriers to the delivery and impact of undergraduate research courses in CPH?
- What are the possible improvements of undergraduate research courses from faculty's point of view?

R1 (opening question)	What are your thoughts the delivery and impact of undergraduate research courses in CPH on students and faculty members?
**Social & Professional role and identity**	• How do you perceive your role as a supervisor for students in undergraduate research?<br>• In your opinion, to which extent do students know about their responsibilities and roles in undergraduate research courses?
**Beliefs about capabilities**	• Are there any research processes that you are more or less confident about performing them in supervising students?<br>• To which extent do you think that the students have the required skills and knowledge to participate in the undergraduate research courses?
R2 (opening question)	What are your thoughts regarding the facilitators and barriers to the delivery and impact of undergraduate Rx courses in CPH?

**Skills**	• In your opinion, what are the most important skills you should possess/develop as a supervisor in Rx course?<br>• What skills and basic knowledge do you believe that students should have to participate in undergraduate research?<br>**Probe: skills that helps in supervising students.

**Beliefs about capabilities**	• Are there any research processes that you are more or less confident about performing them in supervising students?<br>• To which extent do you think that the students have the required skills and knowledge to participate in the undergraduate research courses?
**Beliefs about consequences**	• To what extent do you believe that supervising undergraduate students in conducting research have positive consequences to you as a faculty member?<br>• In your opinion, what are the positive/negative consequences of conducting undergraduate research courses on students, college, and future practice in Qatar?
**Goals:**	What are your primary and secondary goals/aims as a supervisor in undergraduate research courses?
**Environmental and context resources**	To what extent does your working environment at CPH provide the resources needed to perform high quality undergraduate Rx courses?
**Behavioral Regulation**	Describe any regulatory or policy factor that positively or negatively impact the success of the research course?<br>**Probe: conduction of a research project over a semester is inadequate in duration, (feedback on performance, any policy that requires your college administration to give feedback about your performance)
**R3 (opening question)**	What are your thoughts of possible improvements of undergraduate research courses?
**Environmental and context resources**	To what extent does your working environment at CPH provide the resources needed to perform high quality undergraduate Rx courses?
**Behavioral Re gelation**	Describe any regulatory or policy factor that positively or negatively impact the success of the research course?

	**Probe: conduction of a research project over a semester is inadequate in duration, (feedback on performance, Any policy that requires your college administration to give feedback about your performance)

Focus group questions:

1. Opening question for research question 1

What are your thoughts the delivery and impact of undergraduate research courses in CPH on students and faculty members?

- **Social/professional role and identity**

1- How do you perceive your role as a supervisor for students in undergraduate research?

2- In your opinion, to which extent do students know about their responsibilities and roles in undergraduate research courses?

- **Beliefs about capabilities**

3- Are there any research processes that you are more or less confident about performing them in supervising students?

4- To which extent do you think that the students have the required skills and knowledge to participate in the undergraduate research courses?

2. Opening question for research question 2

What are your thoughts regarding the facilitators and barriers to the delivery and impact of undergraduate Rx courses in CPH?

- **Skills**

7- In your opinion, what are the most important skills you should possess/develop as a supervisor in Rx course?

8- What skills and basic knowledge do you believe that students should have to participate in undergraduate research?

Probe: skills that helps in supervising students.

- **Beliefs about capabilities**

- Are there any research processes that you are more or less confident about performing them in supervising students?

- To which extent do you think that the students have the required skills and knowledge to participate in the undergraduate research courses ?

- **Beliefs about consequences**

9- To what extent do you believe that supervising undergraduate students in conducting research Have positive consequences to you as a faculty member?

10- In your opinion, what are the positive/negative consequences of conducting undergraduate research courses on students, college, and future practice in Qatar?

- **Goals:**

11- What are your primary and secondary goals/aims as a supervisor in undergraduate research courses?

- **Environmental context and resources**

12- To what extent does your working environment at CPH provide the resources needed to perform high quality undergraduate Rx courses?

- **Behavioral Regulation**

13- Describe any regulatory or policy factor that positively or negatively impact the success of the research course?

Probe: conduction of a research project over a semester is inadequate in duration, (feedback on performance, Any policy that requires your college administration to give feedback about your performance)

3. **Opening question for research question 3**

What are your thoughts of possible improvements of undergraduate research courses?

- **Environmental context and resources**

- To what extent does your working environment at CPH provide the resources needed to perform high quality undergraduate Rx courses?

- **Behavioral Regulation**

- Describe any regulatory or policy factor that positively or negatively impact the success of the research course?

Probe: conduction of a research project over a semester is inadequate in duration, feedback on performance, any policy that requires your college administration to give feedback about your performance.
